# Pulse pressure and cardiometabolic disease progression: associations with incident multimorbidity and mortality in UK biobank

**DOI:** 10.3389/fcvm.2026.1829003

**Published:** 2026-06-23

**Authors:** Ting Lin, Shuai Hu, Wen Gong, Linqi Li, Zhaowei Zhu, Hebin Xie, Feijun Zhao

**Affiliations:** 1 School of Public Health, Hengyang Medical College, University of South China, Hengyang, Hunan, China; 2 The Affiliated Changsha Central Hospital, Hengyang Medical School, University of South China, Changsha, Hunan, China; 3 Department of Cardiology, The Second Xiangya Hospital of Central South University, Changsha, Hunan, China; 4 Center for Disease Control and Prevention of Mayang, Huaihua, Hunan, China

**Keywords:** all-cause mortality, cardiometabolic diseases, cardiometabolic multimorbidity, pulse pressure, UK biobank

## Abstract

**Background:**

Although pulse pressure (PP) predicts individual cardiometabolic diseases (CMDs), its role in the progression of cardiometabolic multimorbidity (CMM) remains uncertain. This study aimed to investigate the association between PP and CMD progression, from incident CMD to CMM development and all-cause mortality.

**Methods:**

UK Biobank participants (*N* = 403,851) were prospectively assessed. PP was evaluated per 1-standard-deviation (SD) increase and across quartiles (Q1–Q4) using Cox proportional hazards models for associations with: (1) incident CMD, (2) progression to CMM (defined as two or more of type 2 diabetes, coronary heart disease, or stroke), and (3) all-cause mortality. Restricted cubic splines were used to examine nonlinearity, and threshold effects were identified using piecewise regression. Competing-risk analyses (Fine-Gray models) and sensitivity analyses excluding the first 2 years of follow-up were performed to address mortality-related competing events and potential reverse causality, respectively. Subgroup analyses were stratified by age, sex, and body mass index (BMI).

**Results:**

Among 403,851 UK Biobank participants who were free of CMD at baseline, per 1-SD increase in PP was significantly associated with transitions from health to CMD (HR = 1.13, 95% CI: 1.12–1.14) and to CMM (HR = 1.18, 95% CI: 1.15–1.21), with Q4 versus Q1 comparisons indicating 36% higher risks for both outcomes. Notably, the PP–CMM association was strongest among patients with stroke, with an HR of 1.23 (95% CI: 1.11–1.36) per 1-SD increase. Subgroup analyses further showed that this association was most pronounced in participants aged < 60 years, women, and those with BMI 18.5 ≤ 25 kg/m^2^. Threshold-effect analyses identified specific risk turning points: 40 mmHg for incident CMD, 42 mmHg for mortality, and 52 mmHg for CMM development among healthy participants, and 57 mmHg for mortality among participants with established CMM.

**Conclusion:**

Our study demonstrates that elevated PP is significantly associated with higher risks of CMD progression and mortality.

## Graphical Abstract

**Figure:**
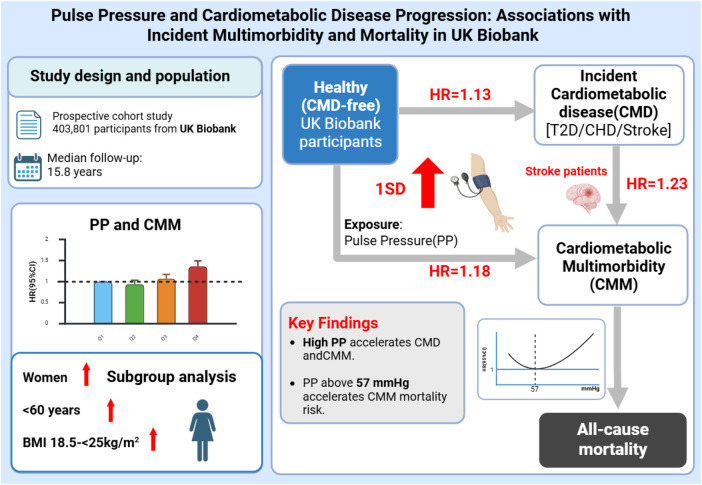

## 1 Introduction

Cardiometabolic diseases (CMDs), a group of chronic conditions that includes coronary heart disease (CHD), stroke, and type 2 diabetes (T2D), are highly prevalent worldwide and represent a major driver of the increasing global disease burden (1). Driven by population aging and lifestyle transitions, the prevalence of CMDs continues to rise, with some individuals progressing to the co-occurrence of multiple CMDs, a condition termed cardiometabolic multimorbidity (CMM) (2). In recent years, the rapidly increasing prevalence of CMM has not only severely compromised patients' health and quality of life but also imposed substantial challenges on healthcare systems worldwide (1, 3, 4). Therefore, in-depth investigation of CMM risk factors and early identification of high-risk populations are critical for developing personalized interventions, improving CMM prevention and control, and alleviating the overall disease burden.

Hypertension independently predicts cardiovascular disease (CVD) risk (5) and accelerates atherosclerosis and vascular endothelial injury, thereby substantially increasing the risks of stroke and CHD (6, 7). It also exacerbates cardiovascular burden in patients with diabetes by promoting insulin resistance and aggravating vascular injury (8, 9). These mechanisms are a major focus of current medical research. However, studies have revealed a complex relationship between blood pressure levels and CVD risk across the full blood pressure range, suggesting that dichotomizing elevated blood pressure may underestimate its true impact on chronic diseases (10). Therefore, comprehensive evaluation of blood pressure metrics in relation to CMDs is clinically important for elucidating pathological mechanisms, optimizing patient management, and reducing cardiovascular event risk.

Pulse pressure (PP), defined as systolic blood pressure (SBP) minus diastolic blood pressure (DBP), is a key indicator of large-artery stiffness and exemplifies such comprehensive metrics. It effectively predicts the risks of CMDs, including diabetes, stroke, and CHD (11–13). Moreover, PP has unique value beyond other blood pressure parameters in predicting CMM, defined as the co-occurrence of two or more CMDs. Elevated PP is a robust independent predictor of incident CHD and stroke in individuals with T2D (14, 15) and has superior predictive power for CHD events compared with other blood pressure indices, such as systolic pressure (16). Notably, the association between PP and CMD appears to be particularly pronounced in diabetic populations (15), a phenomenon potentially linked to arterial stiffness-induced impairment of glucose metabolism (17). Furthermore, widened PP may independently predict all-cause mortality associated with T2D, stroke, and CHD in specific cohorts (18, 19). Therefore, comprehensive investigation of the pathological interplay between PP and CMDs has substantial clinical value for optimizing disease risk stratification.

To our knowledge, the relationships of PP with CMM and CMM-related mortality remain incompletely characterized. To address this knowledge gap, this study used data from more than 400,000 UK Biobank participants to quantify the associations of PP with CMD, CMM, and mortality across diverse baseline disease states. In addition, predefined subgroup analyses by age, sex, and body mass index (BMI), together with sensitivity analyses, were conducted to evaluate the robustness and potential heterogeneity of the findings.

## 2 Materials and methods

### 2.1 Study population

**Figure 1:**
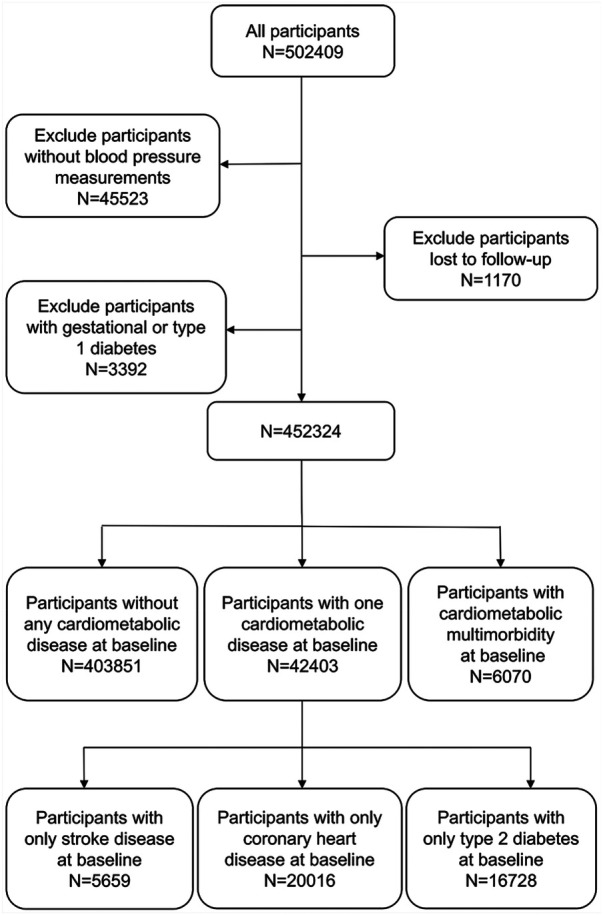
Participant selection flowchart.

Between 2006 and 2010, the UK Biobank cohort recruited more than 500,000 community-dwelling residents aged 40–69 years from 22 assessment centers across the United Kingdom. Participants underwent comprehensive assessments, including touchscreen questionnaires, physical measurements, and biological sample collection (20). In the current analysis, participants were excluded if they met any of the following criteria at baseline: (1) withdrawal from the study (*n* = 1,170), (2) missing systolic or diastolic blood pressure data (*n* = 45,523), or (3) diagnosis of type 1 or gestational diabetes mellitus (*n* = 3,392). The final analytical sample comprised 403,851 individuals without CMD, 42,403 with a single CMD, and 6,070 with CMM at baseline (Figure 1).

### 2.2 Blood pressure measurement

In the UK Biobank, SBP and DBP were measured twice using an Omron 705 IT electronic monitor (fields 4,079 and 4,080), with an interval of at least 1 min between measurements. The mean of the two readings was used for subsequent analyses. PP was calculated as SBP minus DBP.

### 2.3 Ascertainment of cardiometabolic outcomes and mortality

Incident cases of T2D, stroke, and CHD were primarily ascertained using ICD-10 codes (Supplementary Table S1). Supplementary ascertainment sources included self-reported medical conditions (Field 20002), glucose-lowering medication use for T2D (Field 6153), and physician-diagnosed cardiovascular conditions for CHD/stroke ascertainment (Field 6150). All stroke subtypes (ischemic stroke, intracerebral hemorrhage, and subarachnoid hemorrhage) were algorithmically validated through multisource verification (21).

Diagnosis dates were extracted from linked national registries, including Health Episode Statistics (England), Scottish Morbidity Records (Scotland), and the Patient Episode Database (Wales). The date of CMM incidence was defined as the diagnosis date of the second qualifying condition. Mortality data were obtained from death certificates curated by the National Health Service Information Centre (England and Wales) and the National Health Service Central Register (Scotland). The database lock date was 12 December 2024, which served as the censoring date for survival time calculations. The co-primary endpoints were incident CMM and all-cause mortality.

### 2.4 Covariate assessment

Covariates included sociodemographic characteristics, lifestyle factors, and clinical biomarkers:

Sociodemographic characteristics: age (continuous), sex (men/women), educational attainment (categorized as college or university degree, A levels/AS levels or equivalent, O levels/GCSEs or equivalent, CSEs or equivalent, NVQ or HND or HNC or equivalent, and other professional qualifications), and annual household income (£; categories: < 18,000, 18,000–30,999, 31,000–51,999, 52,000–100,000, and > 100,000).

Lifestyle factors: physical activity was quantified using the International Physical Activity Questionnaire (IPAQ) short form. Metabolic equivalent task-minutes per week (MET-min/week) were calculated as 3.3 × walking minutes + 4.0 × moderate activity minutes + 8.0 × vigorous activity minutes and categorized according to IPAQ guidelines as low (< 600), moderate (600–2,999), or high (≥ 3,000) (22). Other lifestyle covariates included smoking status (never/former/current), drinking status (never/former/current), and sleep duration (short: < 6 h; normal: 6–8 h; long: > 8 h) (23).

Clinical factors: BMI was calculated from nurse-measured height and weight and categorized according to WHO criteria (24) as underweight (< 18.5 kg/m^2^), normal weight (18.5–25 kg/m^2^), overweight (25.0–30 kg/m^2^), or obese (≥ 30 kg/m^2^). A supplementary binary grouping was also used: optimal (18.5 ≤ BMI < 25 kg/m^2^) versus non-optimal (< 18.5 or ≥ 25 kg/m^2^). Additional clinical covariates included low-density lipoprotein cholesterol (LDL, continuous), total cholesterol (continuous), glycated hemoglobin (HbA1c, continuous), and family history of CMD (parental or sibling history of heart disease, stroke, or diabetes).

All covariates were ascertained through baseline touchscreen questionnaires or standardized clinical measurements at the initial assessment centers, with detailed protocols documented in the UK Biobank Showcase. Corresponding UK Biobank field codes are provided in Supplementary Table S2.

### 2.5 Statistical Analysis

Baseline characteristics are presented as mean ± standard deviation (SD) for continuous variables and frequencies (percentages) for categorical variables. Follow-up extended from baseline until the earliest occurrence of outcome diagnosis, death, or censoring (12 December 2024). Kaplan–Meier curves stratified by PP quartiles are shown in Supplementary Figure S1. Proportional hazards assumptions were assessed using Schoenfeld residuals (25). Restricted cubic spline curves were fitted to assess nonlinear associations between PP (exposure) and study outcomes (e.g., CMM and all-cause mortality). Nonlinear turning points were identified using piecewise linear regression and verified using likelihood-ratio tests.

Associations of PP, modeled per 1-SD increase and by quartiles [first quartile [Q1] to fourth quartile [Q4]], with CMM and all-cause mortality were estimated using Cox regression. Among participants without CMD at baseline (*n* = 403,851), we examined the associations of PP with (i) incident single CMD, (ii) incident CMM, and (iii) all-cause mortality. Among participants with at least one CMD at baseline (*n* = 42,403), we assessed the associations of PP with incident CMM and mortality. Among participants with prevalent CMM (*n* = 6,070), we evaluated mortality risk.

Hazard ratios (HR) with 95% confidence intervals (CI) are reported per 1-SD increase and for quartile comparisons (reference: Q1). Missing covariate data were imputed using multiple imputation by chained equations (MICE) using random forests (26). The imputation model incorporated age, sex, and BMI as auxiliary variables. Ten imputed datasets were created, with estimates pooled according to Rubin's rules (27). Analyses were conducted using R version 4.4.0. Statistical significance was defined as a two-sided *P* < 0.05.

### 2.6 Subgroup and sensitivity analyses

Given the potential for effect modification by key covariates (e.g., sex and age), we performed subgroup analyses to assess the robustness of the primary findings. Specifically, analyses were stratified by sex (men/women), age [< 60 vs. ≥ 60 years, based on the WHO definition (28)], and BMI category (optimal: 18.5 ≤ BMI < 25 kg/m^2^; non-optimal: < 18.5 or ≥ 25 kg/m^2^).

Two sensitivity analyses were conducted: (a) exclusion of incident cases/deaths within the first 2 years of follow-up to mitigate reverse causation and time-lag bias; and (b) reanalysis of the CMM outcome using the Fine–Gray competing-risks model, with non-cardiovascular death treated as the competing event, to account for the influence of mortality on comorbidity-risk assessment.

## 3 Results

### 3.1 Baseline characteristics and disease transitions

**Figure 2:**
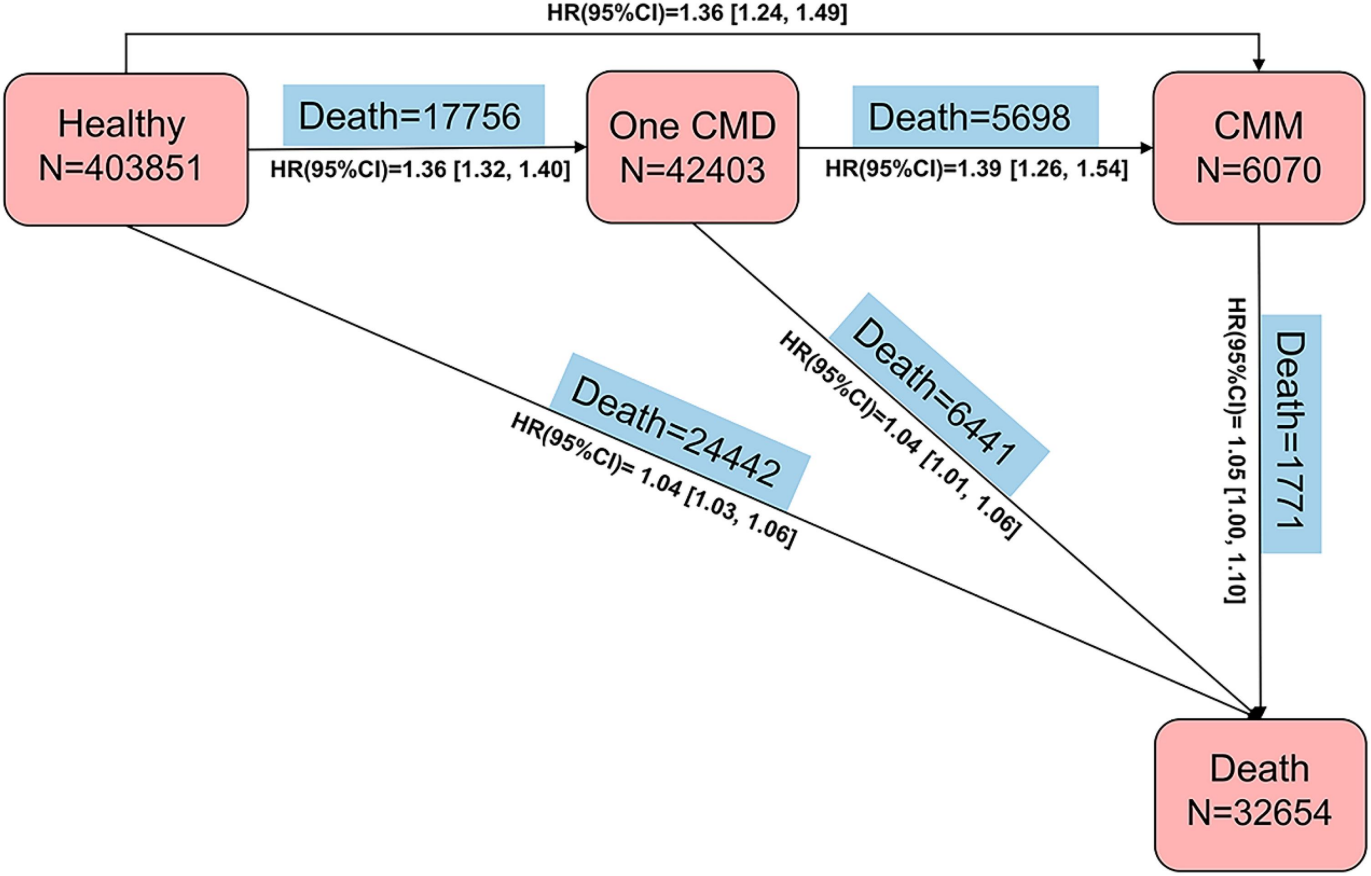
PP-associated risks across cardiometabolic disease transitions. Associations of PP with risks of all transitions from Healthy to one CMD, then to subsequent CMM, and ultimately to all-cause death. HR and 95% CI represent: (1) Q4 vs. Q1 PP comparisons for Healthy→CMD, CMD→CMM, and Healthy→CMM transitions; (2) per 1-SD deviation increase in PP for Healthy→Death, CMD→Death, and CMM→Death transitions. Adjusted for covariates in Model 3.

Table 1 presents the baseline characteristics of all analyzed groups: CMD-free, CMD, CMM, T2D-only, CHD-only, and stroke-only participants, with the latter three groups detailed in Supplementary Table S3. During a median follow-up of 15.8 years, 403,851 participants who were free of CMD at baseline (hereafter referred to as healthy participants or the healthy cohort) were included in this phase of the analysis. Among this cohort, 15,581 participants (3.9%) developed T2D (median time to diagnosis: 7.6 years), 27,477 (6.8%) developed CHD (median time: 7.0 years), 8,353 (2.1%) developed stroke (median time: 7.8 years), and 41,462 (10.3%) developed any CMD (median time: 7.3 years). In addition, 4,858 participants (1.2%) developed CMM (median time: 8.9 years). Before developing CMD, 17,756 participants died; overall, 24,442 participants died during follow-up. The relationships among transition risks between disease states are illustrated in Figure 2.

**Table 1:** Baseline characteristics of CMD-free, CMD, and CMM groups.

Variables	CMD free	CMM	CMD
Age (year)	56.1 (8.1)	62.0 (5.9)	60.5 (6.8)
Men (%)	175914 (43.6)	4390 (72.3)	26537 (62.6)
Ethnicity (%)			
White	382708 (94.8)	5436 (89.6)	38873 (91.7)
Other	21143 (5.2)	634 (10.4)	3530 (8.3)
Educational attainment (%)			
College or University degree	144079 (35.7)	1344 (22.1)	11324 (26.7)
A levels/AS levels or equivalent	53518 (13.3)	745 (12.3)	5297 (12.5)
O levels/GCSEs or equivalent	104125 (25.8)	1552 (25.6)	11087 (26.1)
CSEs or equivalent	35707 (8.8)	782 (12.9)	4621 (10.9)
NVQ or HND or HNC or equivalent	40062 (9.9)	1104 (18.2)	6495 (15.3)
Other professional qualifications	26360 (6.5)	543 (8.9)	3579 (8.4)
Income (%)			
Less than 18,000	88875 (22.0)	3003 (49.5)	15996 (37.7)
18,000–30,999	103181 (25.5)	1660 (27.3)	12063 (28.4)
31,000–51,999	106444 (26.4)	895 (14.7)	8449 (19.9)
52,000–100,000	83086 (20.6)	424 (7.0)	4812 (11.3)
Greater than 100,000	22265 (5.5)	88 (1.4)	1083 (2.6)
Sleep duration (hours/day)	7.1 (1.1)	7.2 (1.6)	7.2 (1.3)
Physical activity (%)			
Moderate	200560 (49.7)	2759 (45.5)	20183 (47.6)
Low	69932 (17.3)	1653 (27.2)	8981 (21.2)
High	133359 (33.0)	1658 (27.3)	13239 (31.2)
Smoking status (%)			
Never	227104 (56.2)	2094 (34.5)	18686 (44.1)
Former	135216 (33.5)	3122 (51.4)	18698 (44.1)
Current	41531 (10.3)	854 (14.1)	5019 (11.8)
Drinking status (%)			
Never	16666 (4.1)	508 (8.4)	2854 (6.7)
Former	12945 (3.2)	610 (10.0)	2541 (6.0)
Current	374240 (92.7)	4952 (81.6)	37008 (87.3)
BMI (%)			
18.5-< 25 kg/m^2^	139906 (34.6)	621 (10.2)	7323 (17.3)
< 18.5 kg/m^2^	2271 (0.6)	13 (0.2)	113 (0.3)
25-< 30 kg/m^2^	172912 (42.8)	2188 (36.0)	17474 (41.2)
≥ 30 kg/m^2^	88762 (22.0)	3248 (53.5)	17493 (41.3)
HbA1c (mmol/L)	35.1 (4.7)	48.1 (13.3)	42.4 (11.8)
LDL (mmol/L)	3.6 (0.8)	2.7 (0.8)	2.9 (0.8)
Cholesterol (mmol/mol)	5.8 (1.1)	4.5 (1.1)	4.8 (1.1)
Family history of CMD (%)			
No family history	141400 (35.0)	1070 (17.6)	9190 (21.7)
Family history present	262451 (65.0)	5000 (82.4)	33213 (78.3)
PP (mmHg)	55.2 (13.4)	60.2 (15.2)	58.7 (14.4)

Data were presented as frequency (%) and mean (standard deviation). CMD cardiometabolic diseases, CMM, cardiometabolic multimorbidity; BMI, body mass index; HbA1c glycated hemoglobin; LDL low-density lipoprotein; PP, pulse pressure.

### 3.2 Association of PP with incident CMM

As shown in Table 2, among participants who were healthy at baseline, each 1-SD increase in PP was significantly and positively associated with CMM risk after multivariable adjustment (Model 3) (HR = 1.18, 95% CI: 1.15–1.21). Participants in the highest quartile (Q4) had a 36% higher risk than those in the lowest quartile (Q1) (HR = 1.36, 95% CI: 1.24–1.49). Analyses stratified by baseline disease status showed similar risk increases among participants with T2D (HR = 1.10, 95% CI: 1.05–1.16) and CHD (HR = 1.12, 95% CI: 1.06–1.18), whereas patients with stroke had a higher risk (HR = 1.23, 95% CI: 1.11–1.36), with Q4 showing an 85% higher risk than Q1 (HR = 1.85, 95% CI: 1.34–2.55). Among participants with baseline CMD, both a per-1-SD increase in PP (HR = 1.15, 95% CI: 1.11–1.19) and the Q4-Q1 comparison (HR = 1.39, 95% CI: 1.26–1.54) demonstrated significant risk elevations.

**Table 2:** Associations of PP with the risk of CMM.

Pulse pressure	Unadjusted model HR (95%CI)	*P*	Model 1 HR (95%CI)	*P*	Model 2 HR (95%CI)	*P*	Model 3 HR (95%CI)	*P*
Healthy participants at baseline (Participants = 403851 / CMM cases = 4858)								
Q1 (lowest)	1 (reference)		1 (reference)		1 (reference)		1 (reference)	
Q2	1.25 [1.13, 1.38]	< 0.001	0.93 [0.85, 1.03]	0.176	0.94 [0.85, 1.04]	0.206	0.94 [0.85, 1.03]	0.194
Q3	1.77 [1.62, 1.94]	< 0.001	1.07 [0.97, 1.17]	0.186	1.06 [0.96, 1.16]	0.250	1.07 [0.97, 1.17]	0.183
Q4 (highest)	2.91 [2.67, 3.17]	< 0.001	1.43 [1.30, 1.56]	< 0.001	1.37 [1.25, 1.50]	< 0.001	1.36 [1.24, 1.49]	< 0.001
Continuous variable	1.47 [1.43, 1.50]	< 0.001	1.20 [1.17, 1.23]	< 0.001	1.18 [1.15, 1.21]	< 0.001	1.18 [1.15, 1.21]	< 0.001
T2D-only participants at baseline (Participants = 16728 / CMM cases = 1738)								
Q1 (lowest)	1 (reference)		1 (reference)		1 (reference)		1 (reference)	
Q2	1.18 [1.02, 1.36]	0.022	1.00 [0.87, 1.16]	0.955	1.02 [0.89, 1.18]	0.752	1.02 [0.89, 1.18]	0.760
Q3	1.26 [1.10, 1.45]	0.001	0.98 [0.85, 1.13]	0.769	0.99 [0.86, 1.15]	0.925	1.00 [0.86, 1.15]	0.965
Q4 (highest)	1.66 [1.45, 1.90]	< 0.001	1.18 [1.03, 1.36]	0.019	1.19 [1.04, 1.38]	0.014	1.19 [1.04, 1.38]	0.014
Continuous variable	1.23 [1.18, 1.28]	< 0.001	1.11 [1.05, 1.16]	< 0.001	1.11 [1.05, 1.16]	< 0.001	1.10 [1.05, 1.16]	< 0.001
Stroke-only participants at baseline (Participants = 5659 / CMM cases = 345)								
Q1 (lowest)	1 (reference)		1 (reference)		1 (reference)		1 (reference)	
Q2	1.11 [0.79, 1.55]	0.560	1.03 [0.73, 1.43]	0.882	1.08 [0.77, 1.52]	0.643	1.13 [0.81, 1.59]	0.471
Q3	1.60 [1.17, 2.19]	0.003	1.41 [1.02, 1.93]	0.036	1.48 [1.08, 2.04]	0.016	1.55 [1.12, 2.14]	0.008
Q4 (highest)	2.01 [1.49, 2.71]	< 0.001	1.68 [1.23, 2.31]	0.001	1.74 [1.26, 2.39]	< 0.001	1.85 [1.34, 2.55]	< 0.001
Continuous variable	1.29 [1.17, 1.41]	< 0.001	1.22 [1.10, 1.35]	< 0.001	1.21 [1.09, 1.34]	< 0.001	1.23 [1.11, 1.36]	< 0.001
CHD-only participants at baseline (Participants = 20016 / CMM cases = 1311)								
Q1 (lowest)	1 (reference)		1 (reference)		1 (reference)		1 (reference)	
Q2	1.05 [0.89, 1.24]	0.577	0.98 [0.83, 1.15]	0.768	1.00 [0.85, 1.18]	0.980	0.99 [0.84, 1.17]	0.934
Q3	1.27 [1.08, 1.48]	0.003	1.13 [0.96, 1.32]	0.137	1.14 [0.97, 1.34]	0.102	1.10 [0.94, 1.29]	0.241
Q4 (highest)	1.48 [1.28, 1.73]	< 0.001	1.30 [1.10, 1.52]	0.001	1.30 [1.11, 1.52]	0.001	1.25 [1.06, 1.47]	0.007
Continuous variable	1.18 [1.12, 1.24]	< 0.001	1.13 [1.07, 1.20]	< 0.001	1.13 [1.07, 1.19]	< 0.001	1.12 [1.06, 1.18]	< 0.001
CMD participants at baseline (Participants = 42403 / CMM cases = 3403)								
Q1 (lowest)	1 (reference)		1 (reference)		1 (reference)		1 (reference)	
Q2	1.14 [1.03, 1.26]	0.014	1.04 [0.93, 1.15]	0.496	1.06 [0.96, 1.18]	0.247	1.08 [0.97, 1.20]	0.139
Q3	1.31 [1.19, 1.44]	< 0.001	1.13 [1.02, 1.25]	0.021	1.14 [1.03, 1.27]	0.010	1.16 [1.04, 1.28]	0.006
Q4 (highest)	1.66 [1.51, 1.83]	< 0.001	1.38 [1.24, 1.52]	< 0.001	1.38 [1.25, 1.53]	< 0.001	1.39 [1.26, 1.54]	< 0.001
Continuous variable	1.22 [1.18, 1.26]	< 0.001	1.15 [1.12, 1.19]	< 0.001	1.15 [1.11, 1.19]	< 0.001	1.15 [1.11, 1.19]	< 0.001

Model 1 was adjusted for age and sex. Model 2 was adjusted for age, sex, educational attainment, sleep duration, smoking status, drinking status, income, physical activity, BMI, and family history of CMD. Model 3 was adjusted for age, sex, educational attainment, sleep duration, smoking status, drinking status, income, physical activity, BMI, family history of CMD, HbA1c, LDL, and cholesterol. The continuous variable represents the effect per standard-deviation increase in pulse pressure. PP, pulse pressure; Q1, first quartile; Q2, second quartile; Q3, third quartile; Q4, fourth quartile; CMM, cardiometabolic multimorbidity; CHD, coronary heart disease; CMD, cardiometabolic diseases; T2D, type 2 diabetes.

### 3.3 PP and risk of incident CMD

Multivariable-adjusted Cox regression (Model 3) showed that, among participants who were healthy at baseline, each 1-SD increase in PP was associated with a 13% higher risk of progression to CMD (HR = 1.13, 95% CI: 1.12–1.14), a 14% higher risk of T2D (HR = 1.14, 95% CI: 1.12–1.15), a 14% higher risk of CHD (HR = 1.14, 95% CI: 1.12–1.15), and a 15% higher risk of stroke (HR = 1.15, 95% CI: 1.12–1.17). Extreme-quartile comparisons (Q4 vs. Q1) demonstrated a 36% increased risk for CMD (HR = 1.36, 95% CI: 1.32–1.40), 35% for T2D (HR = 1.35, 95% CI: 1.28–1.41), 38% for CHD (HR = 1.38, 95% CI: 1.33–1.43), and 35% for stroke (HR = 1.35, 95% CI: 1.26–1.44), with all associations being statistically significant (*P* < 0.05) (Table 3).

**Table 3:** Associations of PP with the risk of CMD.

Pulse pressure	Unadjusted model HR (95%CI)	*P*	Model 1 HR (95%CI)	*P*	Model 2 HR (95%CI)	*P*	Model 3 HR (95%CI)	*P*
CMD as outcome (Participants = 403851 / CMD cases = 41462)								
Q1 (lowest)	1 (reference)		1 (reference)		1 (reference)		1 (reference)	
Q2	1.34 [1.30, 1.38]	< 0.001	1.07 [1.04, 1.11]	< 0.001	1.07 [1.04, 1.11]	< 0.001	1.07 [1.03, 1.10]	< 0.001
Q3	1.74 [1.69, 1.80]	< 0.001	1.18 [1.15, 1.22]	< 0.001	1.17 [1.14, 1.21]	< 0.001	1.18 [1.14, 1.21]	< 0.001
Q4 (highest)	2.41 [2.34, 2.48]	< 0.001	1.40 [1.36, 1.44]	< 0.001	1.36 [1.32, 1.40]	< 0.001	1.36 [1.32, 1.40]	< 0.001
Continuous variable	1.35 [1.34, 1.36]	< 0.001	1.14 [1.13, 1.15]	< 0.001	1.13 [1.12, 1.14]	< 0.001	1.13 [1.12, 1.14]	< 0.001
T2D-only as outcome (Participants = 403851 / T2D cases = 15581)								
Q1 (lowest)	1 (reference)		1 (reference)		1 (reference)		1 (reference)	
Q2	1.19 [1.13, 1.25]	< 0.001	1.02 [0.97, 1.07]	0.508	1.01 [0.96, 1.06]	0.693	0.98 [0.94, 1.04]	0.534
Q3	1.52 [1.45, 1.60]	< 0.001	1.17 [1.11, 1.23]	< 0.001	1.14 [1.09, 1.20]	< 0.001	1.17 [1.11, 1.23]	< 0.001
Q4 (highest)	2.06 [1.96, 2.15]	< 0.001	1.43 [1.36, 1.50]	< 0.001	1.35 [1.28, 1.42]	< 0.001	1.35 [1.28, 1.41]	< 0.001
Continuous variable	1.29 [1.27, 1.31]	< 0.001	1.15 [1.14, 1.17]	< 0.001	1.13 [1.11, 1.15]	< 0.001	1.14 [1.12, 1.15]	< 0.001
CHD-only as outcome (Participants = 403851 / CHD cases = 27477)								
Q1 (lowest)	1 (reference)		1 (reference)		1 (reference)		1 (reference)	
Q2	1.40 [1.35, 1.46]	< 0.001	1.07 [1.03, 1.11]	< 0.001	1.07 [1.03, 1.12]	< 0.001	1.07 [1.03, 1.11]	< 0.001
Q3	1.87 [1.80, 1.94]	< 0.001	1.17 [1.13, 1.22]	< 0.001	1.17 [1.12, 1.21]	< 0.001	1.17 [1.12, 1.21]	< 0.001
Q4 (highest)	2.66 [2.57, 2.76]	< 0.001	1.40 [1.35, 1.46]	< 0.001	1.37 [1.32, 1.43]	< 0.001	1.38 [1.33, 1.43]	< 0.001
Continuous variable	1.39 [1.37, 1.40]	< 0.001	1.15 [1.13, 1.16]	< 0.001	1.14 [1.12, 1.15]	< 0.001	1.14 [1.12, 1.15]	< 0.001
Stroke-only as outcome (Participants = 403851 / Stroke cases = 8353)								
Q1 (lowest)	1 (reference)		1 (reference)		1 (reference)		1 (reference)	
Q2	1.34 [1.24, 1.44]	< 0.001	1.02 [0.94, 1.09]	0.696	1.02 [0.95, 1.10]	0.534	1.03 [0.95, 1.11]	0.480
Q3	1.80 [1.68, 1.93]	< 0.001	1.09 [1.01, 1.17]	0.021	1.10 [1.02, 1.18]	0.012	1.10 [1.02, 1.18]	0.009
Q4 (highest)	2.83 [2.65, 3.02]	< 0.001	1.35 [1.26, 1.44]	< 0.001	1.34 [1.25, 1.43]	< 0.001	1.35 [1.26, 1.44]	< 0.001
Continuous variable	1.44 [1.41, 1.46]	< 0.001	1.15 [1.13, 1.17]	< 0.001	1.14 [1.12, 1.17]	< 0.001	1.15 [1.12, 1.17]	< 0.001

Model 1 was adjusted for age and sex. Model 2 was adjusted for age, sex, educational attainment, sleep duration, smoking status, drinking status, income, physical activity, BMI, and family history of CMD. Model 3 was adjusted for age, sex, educational attainment, sleep duration, smoking status, drinking status, income, physical activity, BMI, family history of CMD, HbA1c, LDL, and cholesterol. The continuous variable represents the effect per standard-deviation increase in pulse pressure. PP, pulse pressure; Q1, first quartile; Q2, second quartile; Q3, third quartile; Q4, fourth quartile; CHD, coronary heart disease; CMD, cardiometabolic diseases; T2D, type 2 diabetes.

### 3.4 PP and all-cause mortality by disease status

As shown in Supplementary Table S4, multivariable-adjusted analysis (Model 3) showed that each 1-SD increase in PP was associated with a 4% higher mortality risk among participants who were healthy at baseline (HR = 1.04, 95% CI: 1.03–1.06), a 9% higher risk among patients with T2D (HR = 1.09, 95% CI: 1.05–1.14), an 8% higher risk among patients with stroke (HR = 1.08, 95% CI: 1.01–1.15), a 4% higher risk among patients with CMD (HR = 1.04, 95% CI: 1.01–1.06), and a 5% higher risk among patients with CMM (HR = 1.05, 95% CI: 1.00–1.10). No significant association was observed between PP and mortality among patients with CHD (*P* ≥ 0.05). Extreme-quartile comparisons (Q4 vs. Q1) showed a 5% higher mortality risk among participants who were healthy at baseline (HR = 1.05, 95% CI: 1.01–1.09), an 18% higher risk among patients with T2D (HR = 1.18, 95% CI: 1.05–1.34), and a 26% higher risk among patients with stroke (HR = 1.26, 95% CI: 1.04–1.54), indicating that PP had the strongest overall mortality association among patients with stroke.

### 3.5 Restricted cubic spline (RCS) and turning point analyses of PP in disease transitions

**Figure 3:**
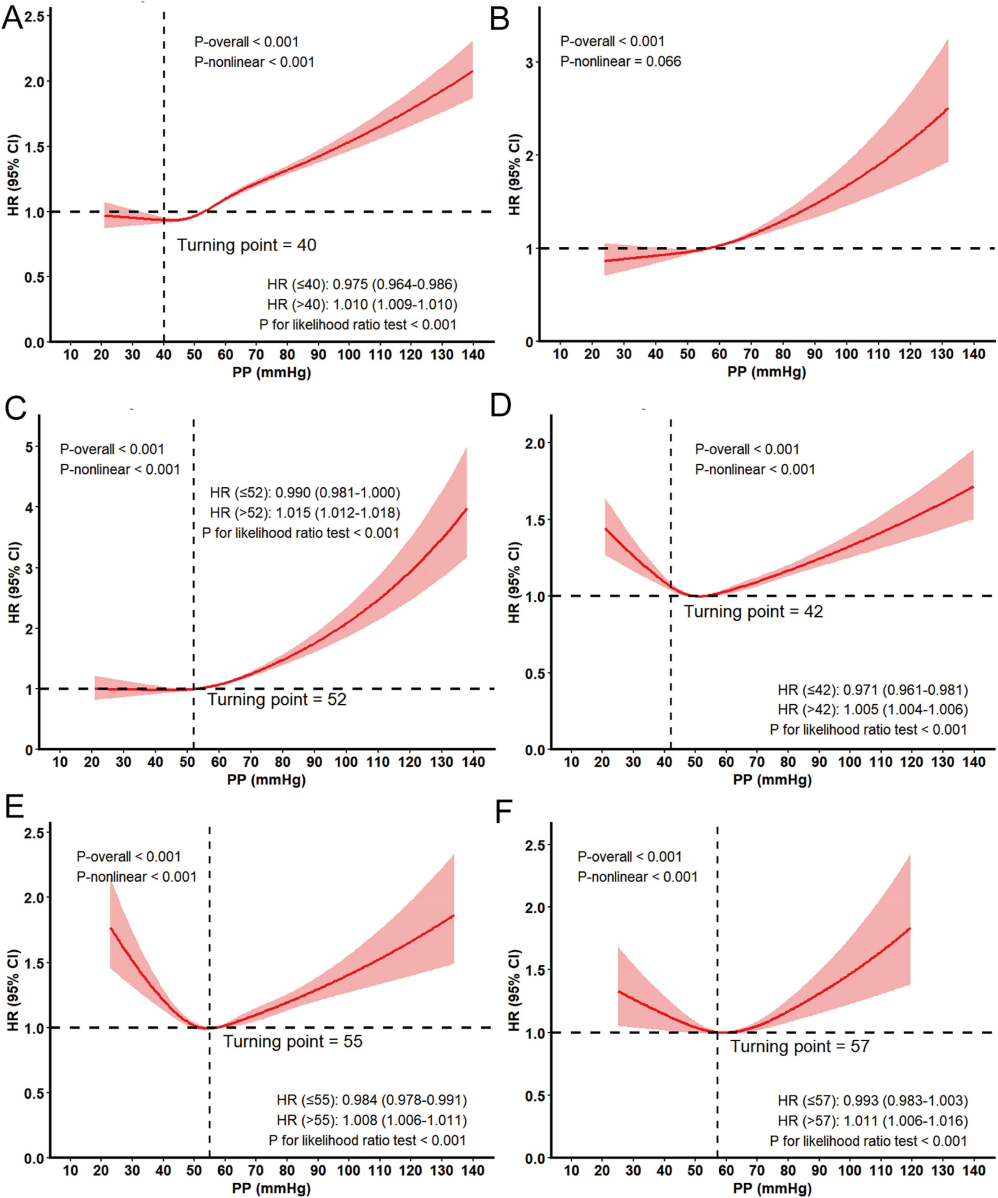
Threshold and spline analyses of PP on disease transitions. Restricted cubic spline curves for PP associations with one CMD, CMM, and all-cause mortality, with turning points identified by piecewise regression. **(A)** Healthy to one CMD; **(B)** CMD to CMM; **(C)** Healthy to CMM; **(D)** Healthy to all-cause mortality; **(E)** one CMD to all-cause mortality; **(F)** CMM to all-cause mortality. Adjusted for covariates in Model 3. CMM, cardiometabolic multimorbidity; CMD, cardiometabolic diseases; PP, pulse pressure.

Using threshold-effect analyses based on piecewise linear regression (Figure 3), we characterized the associations between PP and cardiometabolic disease progression. Among participants who were healthy at baseline, RCS curves revealed significant nonlinear relationships with all outcomes (*P_−nonlinear_* < 0.001). The turning points, representing risk nadirs, were identified at 40 mmHg for CMD transition and 42 mmHg for all-cause mortality. Below these thresholds, higher PP was associated with lower risk; above them, each 1-mmHg increase in PP was associated with a 1.0% increase in CMD risk and a 0.5% increase in mortality risk. For CMM development, the turning point was 52 mmHg, beyond which risk increased more sharply by 1.5% per mmHg. Among participants with one CMD, PP showed a U-shaped association with mortality, with a turning point at 55 mmHg; above this level, each 1-mmHg increase in PP was associated with a 0.8% increase in risk. Similarly, among patients with CMM, the mortality risk turning point was 57 mmHg, above which risk increased by 1.1% per mmHg. In contrast, progression from one CMD to CMM showed a consistent linear increase (*P_−nonlinear_* = 0.066), indicating that risk rose steadily across the observed PP range without a nonlinear turning point.

Supplementary Figure S2 further illustrates these associations across specific conditions. For all-cause mortality, significant nonlinear turning points were identified at 65 mmHg for T2D, 60 mmHg for CHD, and 44 mmHg for stroke. Regarding progression to CMM, a nonlinear turning point was observed at 55 mmHg among patients with T2D, with a 1.3% risk increase per unit above this threshold. Among patients with CHD and stroke, risk increased linearly with rising PP levels (*P_−nonlinear_* = 0.186 and 0.150, respectively), reflecting consistent risk escalation across the observed range.

### 3.6 Subgroup, interaction, and sensitivity analysis results

In subgroup analyses stratified by sex (men/women), age (< 60 vs. ≥ 60 years), and BMI category (optimal: 18.5–25 kg/m^2^; non-optimal: < 18.5 or ≥ 25 kg/m^2^), elevated CMM risk persisted for the highest versus lowest PP quartile (Q4 vs. Q1) among participants without CMD and those with a single CMD (Supplementary Figures S3–S5). Significant multiplicative interactions were observed in the healthy cohort for CMM risk: PP_quartile Q4_ _×_ _age_ (*P_interaction_* = 0.0003), PP_quartile Q4_ _×_ _BMI_ (*P_interaction_* = 0.0008), and PP_quartile Q4_ _×_ _sex_ (*P_interaction_* = 0.0337). The elevation in CMM risk per PP increment was particularly pronounced among participants aged < 60 years, those with optimal BMI, and women.

After exclusion of events occurring within the first 2 years of follow-up, consistent PP–disease associations were observed across all endpoints (Supplementary Table S5), with larger effect estimates suggesting stronger long-term effects of PP on disease progression and mortality. Fine-Gray competing-risk models yielded PP–CMM associations similar to those from standard Cox models, suggesting that mortality events did not substantially alter the observed comorbidity risk (Supplementary Table S6).

## 4 Discussion

Our findings indicate that high PP is positively associated with increased CMM incidence and all-cause mortality among both participants who were healthy at baseline and those with baseline CMD. We also comprehensively assessed transition risks across stages of disease progression, demonstrating significant associations between high PP and incident diabetes, CHD, stroke, or any incident CMD among healthy participants; CMM development among individuals with baseline diabetes, CHD, or stroke; and elevated mortality risk across all cohorts (healthy, baseline CMD, diabetes, and stroke cohorts).

Regarding PP as a predictor of CMD, our results demonstrate that high PP is an independent predictor of stroke, CHD, and diabetes, consistent with existing literature (13, 29, 30). Restricted cubic spline analyses identified turning points that shifted across the disease continuum, specifically 40 mmHg for initial CMD transition and 42 mmHg for mortality among healthy individuals. Notably, the 42-mmHg turning point identified in this study may represent a physiological “watershed” at which risk begins to deviate from the optimal range, providing a sensitive “early-warning” signal for primary prevention. This finding complements the 60-mmHg guideline threshold (31), which may function as an “alarm bell” for established arterial stiffness requiring immediate clinical intervention. Multivariable-adjusted models showed that each 1-SD increment in PP had the strongest association with incident stroke, marginally exceeding its associations with CHD and diabetes. Okada et al. reported a multivariable-adjusted HR of 1.14 (95% CI: 1.05–1.24) for stroke per 1-SD (13.2 mmHg) increase in PP (12), slightly higher than our estimate (HR = 1.10, 95% CI: 1.08–1.13 per 13.3 mmHg). Early Framingham studies established PP as a predictor of CHD risk, with PP superseding systolic and diastolic blood pressure as the strongest predictor in aging populations (13, 32). Regarding diabetes risk, prior reports by Jia et al. suggested sex-specific PP–diabetes associations, with elevated risk observed only in women (30), whereas we observed increased diabetes risk in both sexes, with stronger effects in women. This result is consistent with the findings of Okada et al. (12). Discrepancies may reflect racial/ethnic differences and sample-size variation, warranting further investigation.

Current evidence on PP and incident CMM in healthy populations remains scarce. Existing research has predominantly focused on the predictive capacity of PP for other CVD outcomes among patients with a single CMD, showing superior predictive performance relative to other blood pressure parameters (16, 33, 34). Okada et al. prospectively demonstrated that PP was the strongest predictor of CVD risk among patients with diabetes across all blood pressure metrics examined (PP, mean arterial pressure, systolic pressure, and diastolic pressure) (35). The superior predictive performance of PP cannot be fully explained by conventional blood pressure indicators alone and therefore warrants interpretation from the perspective of vascular physiology. Accordingly, it is important to consider the predictive independence of PP relative to isolated SBP in multimorbidity. Although high PP often results from elevated SBP and reduced DBP, it uniquely captures vascular compliance and arterial elastic recoil—physiological information that SBP alone cannot quantify. Thus, PP may serve as a more sensitive indicator of systemic vascular aging and cumulative mechanical stress, identifying vulnerability before conventional hypertensive criteria are met (36). Our study extends previous work by establishing significant associations between each 1-SD increase in PP and CMM risk among both healthy participants and those with any baseline CMD. Specifically, patients with stroke exhibited a 49% higher CMM risk under high PP (Q4 vs. Q1) than healthy individuals, whereas patients with diabetes and CHD showed marginally lower excess risks. These findings underscore the need for PP monitoring in stroke survivors. Notably, subgroup and interaction analyses revealed amplified CMM risk associated with high PP among women, individuals with optimal BMI, and participants aged < 60 years. This pattern aligns with previous reports on CMM risk in CMD cohorts. Kannel et al. identified greater susceptibility to diabetic CVD in women than in men (37). Sattar et al. reported elevated cardiovascular and mortality risks among patients diagnosed with T2D at younger ages (38). A meta-analysis by Zaccardi et al. demonstrated increased cardiovascular mortality among individuals with diabetes and BMI < 27 kg/m^2^ (39). Given the sparse representation of underweight individuals (BMI < 18.5 kg/m^2^) in our cohort, we adopted WHO-based BMI categories: optimal (18.5–25 kg/m^2^) and non-optimal (< 18.5 or ≥ 25 kg/m^2^). The quantitative interplay among BMI, PP, and CMM risk warrants further investigation.

Significant associations were observed between elevated PP and all-cause mortality, particularly among patients with established CMM. Subgroup analyses suggested a protective association against CMM incidence when PP was maintained within quartiles 2–3 (vs. Q1) among individuals aged > 60 years. Although no significant sex- or BMI-based differences in PP–mortality associations were detected, restricted cubic spline analyses identified a critical risk turning point of 57 mmHg for all-cause mortality among patients with established CMM. These findings indicate that PP below this threshold is longitudinally associated with lower mortality risk, providing a potential clinical reference for risk stratification in older adults. Further analyses of mortality among participants with a single CMD demonstrated that PP predicted all-cause death in patients with T2D and stroke, confirming prior evidence (18, 19). Notably, we observed no association between PP and mortality among patients with CHD, a finding that warrants further confirmation.

The association between elevated PP and CMM may arise from a bidirectional vicious cycle in which arterial stiffness-induced pathological hemodynamic stress and metabolic dysregulation (e.g., insulin resistance and dyslipidemia) mutually exacerbate vascular damage and metabolic dysfunction (17, 40), further accelerating arterial stiffening. Notably, this relationship showed marked heterogeneity: women, individuals with optimal BMI (18.5–25 kg/m^2^), and those aged < 60 years had significantly higher CMM risk at elevated PP than men, individuals with non-optimal BMI, and older adults. This variability may be partly driven by postmenopausal estrogen decline, which can impair vascular–metabolic homeostasis in women (41). Among individuals aged < 60 years, this heightened risk likely reflects early vascular aging (EVA) (42), whereby premature arterial stiffening and longer cumulative exposure to pulsatile stress accelerate the transition to CMM. The heightened risk observed in the optimal-BMI population may suggest a “hidden vascular aging” phenotype, in which arterial stiffening progresses despite the absence of traditional metabolic indicators such as obesity. Alternatively, it may reflect the “obesity paradox,” a well-documented epidemiological phenomenon in which normal-weight individuals sometimes have higher vascular risk than overweight counterparts under the same hemodynamic conditions; increased adipose tissue may provide hemodynamic buffering or metabolic reserve, whereas normal-weight individuals with elevated PP may harbor intrinsic vascular vulnerabilities that accelerate transition to CMM (43). In addition to BMI, glycemic control is a critical synergistic factor. As shown in Table 1, HbA1c levels were substantially higher in the CMM group (48.1 mmol/mol) than in other disease-stage groups. Hyperglycemia promotes the formation of advanced glycation end-products (AGEs) in the vessel wall, directly facilitating collagen-fiber cross-linking and thereby increasing vascular stiffness and widening PP. Thus, integrated management of both glucose and PP is essential for disrupting this pathogenic cascade (44). A JAMA study demonstrated graded mortality risks with increasing CMD burden, with HRs of approximately 2, 4, and 8 for single-CMD, two-CMD, and three-CMD combinations, respectively (2). These findings support public health integration of algorithm-derived PP metrics through automated calculation from routine blood pressure monitoring to enable risk-stratified screening, with priority given to women, individuals aged < 60 years, and those with optimal BMI. This framework also supports precision interventions, including dual-pathway vascular–metabolic optimization for prevention in high-risk subgroups and concurrent PP control, glycemic regulation, and lipid management in patients with CMM, collectively helping to disrupt pathogenic cascades, extend survival, and reduce healthcare expenditure.

The primary strength of this study lies in its use of the large-scale UK Biobank cohort with prolonged follow-up to systematically evaluate baseline PP across the entire CMM continuum, from initial health status through CMM onset to all-cause mortality. To our knowledge, this is the first study to establish PP as an independent predictor of mortality among individuals with prevalent CMM. Several limitations should be acknowledged. First, generalizability may be restricted because the cohort predominantly comprised individuals of White British ancestry, indicating the need for multiethnic validation. Second, residual confounding may persist despite robust sensitivity analyses and adjustment for key cardiometabolic risk factors. Third, baseline non-CMD comorbidities (e.g., cancer) may have influenced PP or introduced competing risks, although this concern was mitigated by excluding early events and applying competing-risk models. Finally, PP was measured at a single time point, precluding assessment of longitudinal hemodynamic changes; although this limitation was partly offset by the large cohort size, confirmation using repeated measurements is warranted. These constraints require cautious interpretation and highlight future priorities, including multiethnic replication, enhanced confounder control, comorbidity-specific subgroup analyses, and integration of ambulatory blood pressure monitoring.

## 5 Conclusion

This study shows that elevated PP is associated with CMD/CMM progression and mortality. Threshold-effect analyses identified shifting risk turning points: 40 mmHg for incident CMD, 52 mmHg for CMM development, and 57 mmHg for mortality among individuals with established CMM. These stage-specific thresholds provide actionable evidence for clinical risk stratification, particularly in high-risk subgroups such as women and younger adults.

## Data Availability

The data analyzed in this study is subject to the following licenses/restrictions: Data must be applied for and accessed through the official UK Biobank website following approval of a research proposal. Requests to access these datasets should be directed to https://biobank.ndph.ox.ac.uk/showcase/download.cgi.
