# Supplementary material for: Pulse pressure and cardiometabolic disease progression: associations with incident multimorbidity and mortality in UK biobank
Source: Front Cardiovasc Med. 2026 Jun 23;13:1829003. doi: 10.3389/fcvm.2026.1829003 (PMC13348076; doi:10.3389/fcvm.2026.1829003)
Supplement: Supplementary file 1 [file Datasheet1.pdf]

Supplementary File

Pulse Pressure and Cardiometabolic Disease Progression: Associations with Incident Multimorbidity and Mortality in UK Biobank

Table S1. Definitions of the three cardiometabolic diseases 1

Table S2. Additional UK Biobank Field Codes for Covariates 2

Table S3. Baseline characteristics of single-disease subgroups 3

Table S4. Associations of PP with the risk of death 4

Table S5. PP disease associations in sensitivity analysis excluding first two years 6

Table S6. PP-CMM associations from fine-gray competing risk models 7

Figure S1. PP-associated incident CMD, CMM, and mortality: Kaplan-Meier curves by PP quartiles. 8

Figure S2. PP-associated transition from T2D/CHD/Stroke to CMM or mortality: RCS. 9

Figure S3. Subgroup analysis of PP-CMD/CMM-mortality associations by sex subgroups 10

Figure S4. Subgroup analysis of PP-CMD/CMM-mortality associations by age subgroups ... 11

Figure S5. Subgroup analysis of PP-CMD/CMM-mortality associations by BMI category. 12

Table S1. Definitions of the three cardiometabolic diseases

	Fields	Codes
Type 2 diabetes	41270	E11
	20002	1223
	2443	1
	6153	3
	6177	3
	2976	-
Coronary heart disease	41270	I20, I21, I22, I23, I24, I25
	20002	1066,1074,1075
	6150	1, 2
	3627	-
	3894	-
Stroke	41270	I60, I61, I62, I63, I64, I69
	20002	1081, 1086, 1491, 1583
	6150	3
	4056	-
	42006	-
	42008	-
	42010	-
	42012	-

Table S2. Additional UK Biobank Field Codes for Covariates

Variable	Fields
Age	21022
Sex	31
Ethnicity	21000
Educational attainment	6138
Income	738
Sleep duration	1160
Physical activity	22040
Smoking status	20116
Drinking status	20117
Body mass index	21001
Glycated hemoglobin (HbA1c)	30750
LDL	30780
Cholesterol	30690
Illnesses of father Family history	20107
Illnesses of mother Family history	20110
Illnesses of siblings Family history	20111
Systolic blood pressure	4080
Diastolic blood pressure	4079
Date of attending assessment centre	53
Type 1 diabetes	20002, 41270
Gestational diabetes	4041, 20002
participants lost to follow-up	190
Date of death	40000
Date E11 first reported	130708
Date I20 first reported	131296
Date I21 first reported	131298
Date I22 first reported	131300
Date I23 first reported	131302
Date I24 first reported	131304
Date I25 first reported	131306
Date I60 first reported	131360
Date I61 first reported	131362
Date I62 first reported	131364
Date I63 first reported	131366
Date I64 first reported	131368
Date I69 first reported	131378

Table S3. Baseline characteristics of single-disease subgroups

	T2D	Stroke	CHD
Age (year)	59.4 (7.1)	60.0 (7.1)	61.5 (6.3)
Male (%)	10188 (60.9)	3044 (53.8)	13305 (66.5)
Ethnicity (%)			
White	14412 (86.2)	5427 (95.9)	19034 (95.1)
Other	2316 (13.8)	232 (4.1)	982 (4.9)
Educational attainment (%)			
College or University degree	4650 (27.8)	1477 (26.1)	5197 (26.0)
A levels/AS levels or equivalent	2155 (12.9)	705 (12.5)	2437 (12.2)
O levels/GCSEs or equivalent	4208 (25.2)	1600 (28.3)	5279 (26.4)
CSEs or equivalent	1919 (11.5)	630 (11.1)	2072 (10.4)
NVQ or HND or HNC or equivalent	2460 (14.7)	781 (13.8)	3254 (16.3)
Other professional qualifications	1336 (8.0)	466 (8.2)	1777 (8.9)
Income (%)			
Less than 18,000	6075 (36.3)	2185 (38.6)	7736 (38.6)
18,000 to 30,999	4779 (28.6)	1588 (28.1)	5696 (28.5)
31,000 to 51,999	3418 (20.4)	1116 (19.7)	3915 (19.6)
52,000 to 100,000	2035 (12.2)	623 (11.0)	2154 (10.8)
Greater than 100,000	421 (2.5)	147 (2.6)	515 (2.6)
Sleep duration (hours/day)	7.2 (1.3)	7.2 (1.4)	7.2 (1.3)
Physical activity (%)			
Moderate	8033 (48.0)	2668 (47.1)	9482 (47.4)
Low	3916 (23.4)	1169 (20.7)	3896 (19.5)
High	4779 (28.6)	1822 (32.2)	6638 (33.2)
Smoking status (%)			
Never	8002 (47.8)	2546 (45.0)	8138 (40.7)
Former	6945 (41.5)	2268 (40.1)	9485 (47.4)
Current	1781 (10.6)	845 (14.9)	2393 (12.0)
Drinking status (%)			
Never	1482 (8.9)	304 (5.4)	1068 (5.3)
Former	1086 (6.5)	387 (6.8)	1068 (5.3)
Current	14160 (84.6)	4968 (87.8)	17880 (89.3)
BMI (kg/m^2)	3.4 (0.7)	3.0 (0.8)	3.1 (0.7)
HbA1c (mmol/L)	50.8 (13.6)	36.4 (8.1)	37.1 (5.0)
LDL (mmol/L)	2.8 (0.8)	3.1 (0.9)	3.0 (0.8)
Cholesterol (mmol/mol)	4.6 (1.1)	5.1 (1.2)	4.9 (1.1)
Family history of CMD (%)			
No family history	3534 (21.1)	1524 (26.9)	4132 (20.6)
Family history present	13194 (78.9)	4135 (73.1)	15884 (79.4)
PP (mmHg)	59.3 (14.3)	57.4 (14.4)	58.6 (14.4)

Data were presented as frequency (%) and mean (standard deviation). Abbreviations: CMD cardiometabolic diseases, CMM cardiometabolic multimorbidity, BMI body mass index, HbA1c glycated hemoglobin, LDL low-density lipoprotein, PP pulse pressure, CHD coronary heart disease, T2D type 2 diabetes.

Table S4. Associations of PP with the risk of death

Pulse pressure	Unadjusted model HR (95%CI)	*P*	Model 1 HR (95%CI)	*P*	Model 2 HR (95%CI)	*P*	Model 3 HR (95%CI)	P
**CMM participants at baseline (Participants=6070 / death cases=1771)**								
Q1 (lowest)	1 (reference)		1 (reference)		1 (reference)		1 (reference)	
Q2	1.00 [0.87, 1.14]	0.941	0.89 [0.78, 1.02]	0.103	0.92 [0.80, 1.05]	0.213	0.91 [0.80, 1.05]	0.203
Q3	1.09 [0.96, 1.25]	0.190	0.90 [0.79, 1.04]	0.147	0.89 [0.78, 1.03]	0.114	0.89 [0.78, 1.02]	0.098
Q4 (highest)	1.43 [1.26, 1.63]	< 0.001	1.10 [0.97, 1.26]	0.143	1.10 [0.96, 1.25]	0.181	1.08 [0.95, 1.24]	0.235
Continuous variable	1.16 [1.11, 1.21]	< 0.001	1.06 [1.01, 1.11]	0.016	1.06 [1.01, 1.11]	0.022	1.05 [1.00, 1.10]	0.039
**CMD participants at baseline (Participants=42403 / death cases=6641)**								
Q1 (lowest)	1 (reference)		1 (reference)		1 (reference)		1 (reference)	
Q2	1.03 [0.96, 1.11]	0.453	0.87 [0.80, 0.93]	< 0.001	0.90 [0.84, 0.97]	0.005	0.90 [0.83, 0.97]	0.004
Q3	1.18 [1.10, 1.26]	< 0.001	0.88 [0.82, 0.95]	< 0.001	0.91 [0.85, 0.98]	0.012	0.91 [0.84, 0.98]	0.008
Q4 (highest)	1.52 [1.42, 1.62]	< 0.001	1.03 [0.96, 1.11]	0.354	1.05 [0.98, 1.13]	0.165	1.04 [0.97, 1.12]	0.246
Continuous variable	1.19 [1.16, 1.21]	< 0.001	1.04 [1.02, 1.07]	0.001	1.04 [1.02, 1.07]	0.001	1.04 [1.01, 1.06]	0.003
**T2D-only participants at baseline (Participants=16728 / death cases=2438)**								
Q1 (lowest)	1 (reference)		1 (reference)		1 (reference)		1 (reference)	
Q2	1.17 [1.03, 1.32]	0.016	0.95 [0.83, 1.07]	0.380	0.98 [0.87, 1.11]	0.776	0.98 [0.86, 1.11]	0.733
Q3	1.36 [1.21, 1.53]	< 0.001	0.97 [0.86, 1.09]	0.598	1.00 [0.89, 1.14]	0.940	1.00 [0.89, 1.14]	0.941
Q4 (highest)	1.85 [1.65, 2.07]	< 0.001	1.17 [1.03, 1.31]	0.012	1.19 [1.05, 1.34]	0.006	1.18 [1.05, 1.34]	0.006
Continuous variable	1.27 [1.22, 1.31]	< 0.001	1.09 [1.05, 1.14]	< 0.001	1.09 [1.05, 1.14]	< 0.001	1.09 [1.05, 1.14]	< 0.001
**CHD-only participants at baseline (Participants=20016 / death cases=3279)**								
Q1 (lowest)	1 (reference)		1 (reference)		1 (reference)		1 (reference)	
Q2	0.92 [0.83, 1.02]	0.106	0.79 [0.72, 0.87]	< 0.001	0.82 [0.74, 0.91]	< 0.001	0.82 [0.74, 0.91]	< 0.001
Q3	0.98 [0.89, 1.08]	0.697	0.76 [0.69, 0.84]	< 0.001	0.77 [0.70, 0.85]	< 0.001	0.77 [0.69, 0.85]	< 0.001
Q4 (highest)	1.28 [1.17, 1.41]	< 0.001	0.93 [0.84, 1.02]	0.117	0.94 [0.85, 1.04]	< 0.001	0.93 [0.85, 1.03]	0.169
Continuous variable	1.11 [1.08, 1.15]	< 0.001	1.00 [0.96, 1.03]	0.858	1.00 [0.96, 1.03]	0.811	0.99 [0.96, 1.03]	0.690
**Stroke-only participants at baseline (Participants=5659 / death cases=924)**								
Q1 (lowest)	1 (reference)		1 (reference)		1 (reference)		1 (reference)	
Q2	1.09 [0.89, 1.33]	0.415	0.94 [0.76, 1.15]	0.529	1.01 [0.82, 1.24]	0.938	1.01 [0.82, 1.24]	0.928
Q3	1.49 [1.23, 1.81]	< 0.001	1.12 [0.92, 1.36]	0.265	1.18 [0.97, 1.43]	0.102	1.18 [0.97, 1.44]	0.098
Q4 (highest)	1.85 [1.54, 2.23]	< 0.001	1.25 [1.03, 1.51]	0.026	1.26 [1.04, 1.53]	0.019	1.26 [1.04, 1.54]	0.018
Continuous variable	1.24 [1.17, 1.32]	< 0.001	1.09 [1.02, 1.16]	0.009	1.08 [1.01, 1.15]	0.025	1.08 [1.01, 1.15]	0.024
**Healthy participants at baseline (Participants=403851 / death cases=24442)**								
Q1 (lowest)	1 (reference)		1 (reference)		1 (reference)		1 (reference)	
Q2	1.25 [1.20, 1.30]	< 0.001	0.93 [0.89, 0.97]	< 0.001	0.96 [0.92, 1.00]	0.033	0.96 [0.92, 1.00]	0.057
Q3	1.60 [1.54, 1.67]	< 0.001	0.94 [0.90, 0.98]	0.001	0.97 [0.93, 1.01]	0.094	0.97 [0.93, 1.01]	0.146
Q4 (highest)	2.28 [2.20, 2.37]	< 0.001	1.03 [0.99, 1.07]	0.156	1.05 [1.01, 1.09]	0.020	1.05 [1.01, 1.09]	0.013
Continuous variable	1.34 [1.33, 1.36]	< 0.001	1.04 [1.03, 1.05]	< 0.001	1.04 [1.03, 1.06]	< 0.001	1.04 [1.03, 1.06]	< 0.001

Model 1 was adjusted for age and sex; Model 2 was adjusted for age, sex, educational attainment, Sleep duration, smoking status, drinking status, Income, physical activity, BMI and family history of CMD; Model 3 was adjusted for age, sex, educational attainment, Sleep duration, smoking status, drinking status, Income, physical activity, BMI, family history of CMD, HbA1c, LDL and Cholesterol. Continuous variable was represented by per standard deviation increase of pulse pressure. Abbreviations: Q1 the first quartile, Q2 the second quartile, Q3 the third quartile, Q4 the fourth quartile, CMM cardiometabolic multimorbidity, CHD coronary heart disease, CMD cardiometabolic diseases, T2D type 2 diabetes, PP pulse pressure.

Table S5. PP disease associations in sensitivity analysis excluding first two years

Pulse pressure	Unadjusted model HR (95%CI)	*P*	Adjusted model HR (95%CI)	*P*
**Healthy participants at baseline (Participants=402166 / CMM cases=4706)**				
Q1 (lowest)	1 (reference)		1 (reference)	
Q2	1.26 [1.14, 1.39]	< 0.0001	0.95 [0.86, 1.05]	0.3034
Q3	1.76 [1.61, 1.93]	< 0.0001	1.07 [0.97, 1.17]	0.1937
Q4 (highest)	2.90 [2.67, 3.16]	< 0.0001	1.37 [1.24, 1.50]	< 0.0001
Continuous variable	1.46 [1.43, 1.50]	< 0.0001	1.18 [1.15, 1.22]	< 0.0001
**CMD participants at baseline (Participants=41727 / CMM cases=3181)**				
Q1 (lowest)	1 (reference)		1 (reference)	
Q2	1.14 [1.03, 1.27]	0.0158	1.09 [0.97, 1.21]	0.1407
Q3	1.33 [1.20, 1.47]	< 0.0001	1.17 [1.05, 1.30]	0.0035
Q4 (highest)	1.69 [1.53, 1.86]	< 0.0001	1.42 [1.28, 1.57]	< 0.0001
Continuous variable	1.23 [1.19, 1.27]	< 0.0001	1.15 [1.11, 1.19]	< 0.0001
**CMM participants at baseline (Participants=5922 / death cases=1623)**				
Q1 (lowest)	1 (reference)		1 (reference)	
Q2	1.02 [0.88, 1.18]	0.780	0.93 [0.81, 1.08]	0.3455
Q3	1.15 [1.00, 1.32]	0.056	0.93 [0.80, 1.07]	0.3142
Q4 (highest)	1.49 [1.30, 1.71]	< 0.0001	1.12 [0.97, 1.29]	0.1212
Continuous variable	1.17 [1.12, 1.23]	< 0.0001	1.06 [1.01, 1.12]	0.0197
**Healthy participants at baseline (Participants=402305 / death cases=22896)**				
Q1 (lowest)	1 (reference)		1 (reference)	
Q2	1.27 [1.21, 1.32]	< 0.0001	0.97 [0.93, 1.01]	0.1573
Q3	1.64 [1.57, 1.70]	< 0.0001	0.98 [0.94, 1.02]	0.4138
Q4 (highest)	2.34 [2.25, 2.43]	< 0.0001	1.07 [1.02, 1.11]	0.0024
Continuous variable	1.35 [1.34, 1.37]	< 0.0001	1.05 [1.03, 1.06]	0.0001
**CMD participants at baseline (Participants=41933 / death cases=6171)**				
Q1 (lowest)	1 (reference)		1 (reference)	
Q2	1.03 [0.96, 1.11]	0.413	0.90 [0.83, 0.97]	0.0054
Q3	1.21 [1.13, 1.30]	< 0.0001	0.93 [0.86, 1.00]	0.0451
Q4 (highest)	1.56 [1.46, 1.67]	< 0.0001	1.06 [0.99, 1.14]	0.1064
Continuous variable	1.20 [1.17, 1.23]	< 0.0001	1.05 [1.02, 1.07]	0.0005

Adjusted model was adjusted for age, sex, educational attainment, Sleep duration, smoking status, drinking status, Income, physical activity, BMI, family history of CMD, HbA1c, LDL and Cholesterol. Continuous variable was represented by per standard deviation increase of pulse pressure. Abbreviations: Q1 the first quartile, Q2 the second quartile, Q3 the third quartile, Q4 the fourth quartile, CMM cardiometabolic multimorbidity, CMD cardiometabolic diseases, PP pulse pressure.

Table S6. PP-CMM associations from fine-gray competing risk models

Pulse pressure	Unadjusted model HR (95%CI)	*P*	Adjusted model HR (95%CI)	*P*
**Healthy participants at baseline (Participants=403851 / CMM cases=4858)**				
Q1 (lowest)	1 (reference)		1 (reference)	
Q2	1.24 [1.12, 1.36]	< 0.0001	0.95 [0.86, 1.04]	0.27
Q3	1.73 [1.58, 1.90]	< 0.0001	1.08 [0.98, 1.19]	0.12
Q4 (highest)	2.77 [2.54, 3.01]	< 0.0001	1.36 [1.23, 1.49]	< 0.0001
Continuous variable	1.44 [1.41, 1.47]	< 0.0001	1.18 [1.14, 1.21]	< 0.0001
**CMD participants at baseline (Participants=42403 / CMM cases=3403)**				
Q1 (lowest)	1 (reference)		1 (reference)	
Q2	1.15 [1.04, 1.27]	0.0086	1.11 [1.00, 1.23]	0.052
Q3	1.29 [1.17, 1.42]	< 0.0001	1.18 [1.07, 1.31]	0.0016
Q4 (highest)	1.57 [1.43, 1.73]	< 0.0001	1.39 [1.25, 1.54]	< 0.0001
Continuous variable	1.19 [1.16, 1.23]	< 0.0001	1.14 [1.11, 1.19]	< 0.0001
**T2D-only participants at baseline (Participants=16728 / CMM cases=1738)**				
Q1 (lowest)	1 (reference)		1 (reference)	
Q2	1.16 [1.01, 1.34]	0.034	1.03 [0.90, 1.19]	0.69
Q3	1.24 [1.08, 1.42]	0.0024	1.02 [0.88, 1.18]	0.80
Q4 (highest)	1.53 [1.34, 1.75]	< 0.0001	1.16 [1.01, 1.35]	0.043
Continuous variable	1.19 [1.14, 1.25]	< 0.0001	1.09 [1.04, 1.15]	< 0.0001
**CHD-only participants at baseline (Participants=20016 / CMM cases=1311)**				
Q1 (lowest)	1 (reference)		1 (reference)	
Q2	1.06 [0.90, 1.24]	0.49	1.03 [0.88, 1.22]	0.7
Q3	1.29 [1.10, 1.50]	0.0015	1.18 [1.00, 1.39]	0.047
Q4 (highest)	1.43 [1.23, 1.67]	< 0.0001	1.30 [1.10, 1.53]	0.0019
Continuous variable	1.16 [1.10, 1.22]	< 0.0001	1.13 [1.07, 1.19]	< 0.0001
**Stroke-only participants at baseline (Participants=5659 / CMM cases=354)**				
Q1 (lowest)	1 (reference)		1 (reference)	
Q2	1.14 [0.82, 1.59]	0.43	1.17 [0.84, 1.64]	0.3600
Q3	1.48 [1.09, 2.03]	0.013	1.48 [1.06, 2.06]	0.0220
Q4 (highest)	1.85 [1.37, 2.50]	< 0.0001	1.81 [1.30, 2.53]	0.0005
Continuous variable	1.24 [1.13, 1.37]	< 0.0001	1.22 [1.10, 1.36]	0.0002

Adjusted model was adjusted for age, sex, educational attainment, Sleep duration, smoking status, drinking status, Income, physical activity, BMI, family history of CMD, HbA1c, LDL and Cholesterol. Continuous variable was represented by per standard deviation increase of pulse pressure. Abbreviations: Q1 the first quartile, Q2 the second quartile, Q3 the third quartile, Q4 the fourth quartile, CMM cardiometabolic multimorbidity, CHD coronary heart disease, CMD cardiometabolic diseases, T2D type 2 diabetes, PP pulse pressure.

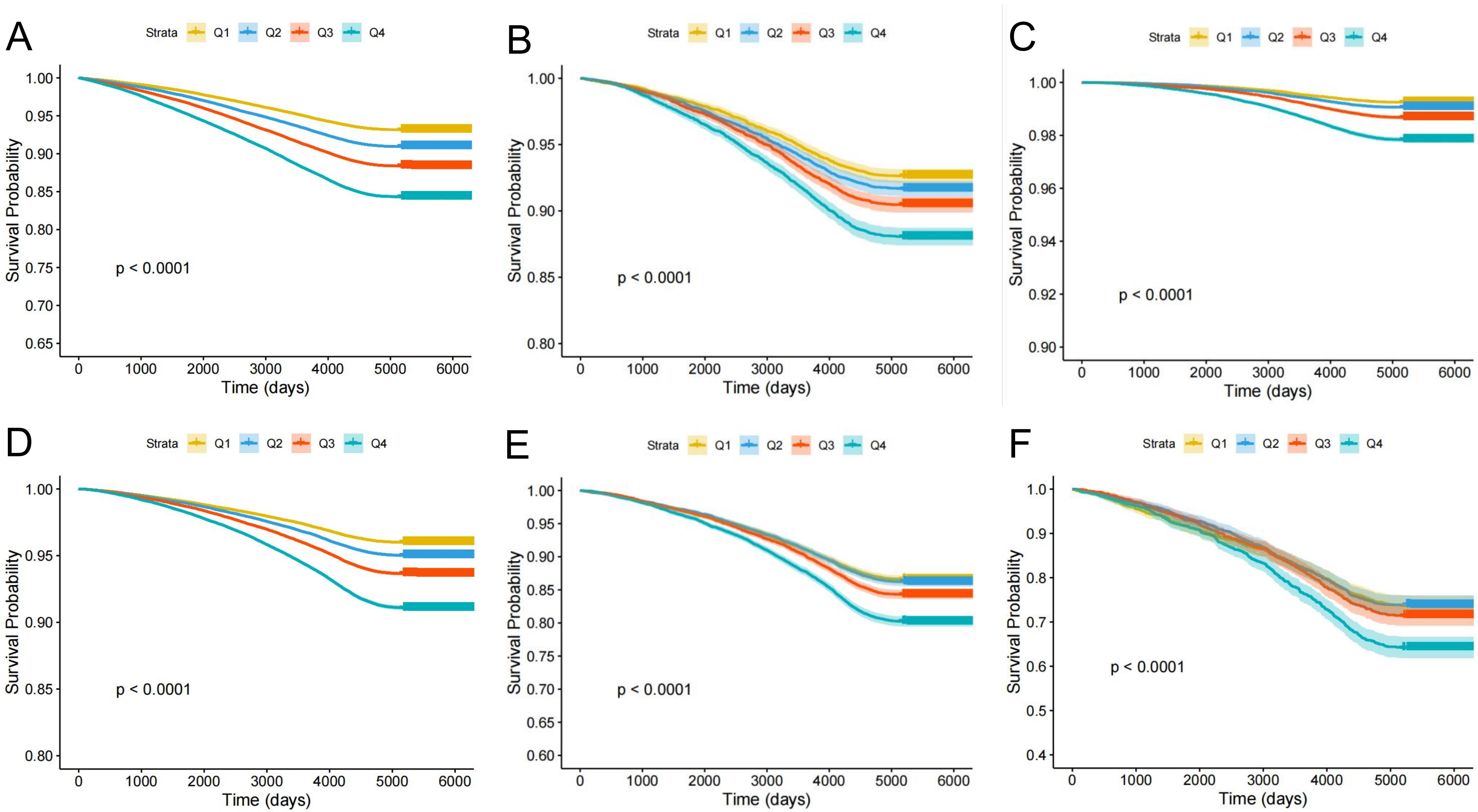

Figure S1. PP-associated incident CMD, CMM, and mortality: Kaplan-Meier curves by PP quartiles.

(A) Healthy to one CMD; (B) one CMD to CMM; (C) Healthy to CMM; (D) Healthy to all-cause mortality; (E) one CMD to all-cause mortality; (F) CMM to all-cause mortality. Abbreviations: CMM cardiometabolic multimorbidity, CMD cardiometabolic diseases, PP pulse pressure.

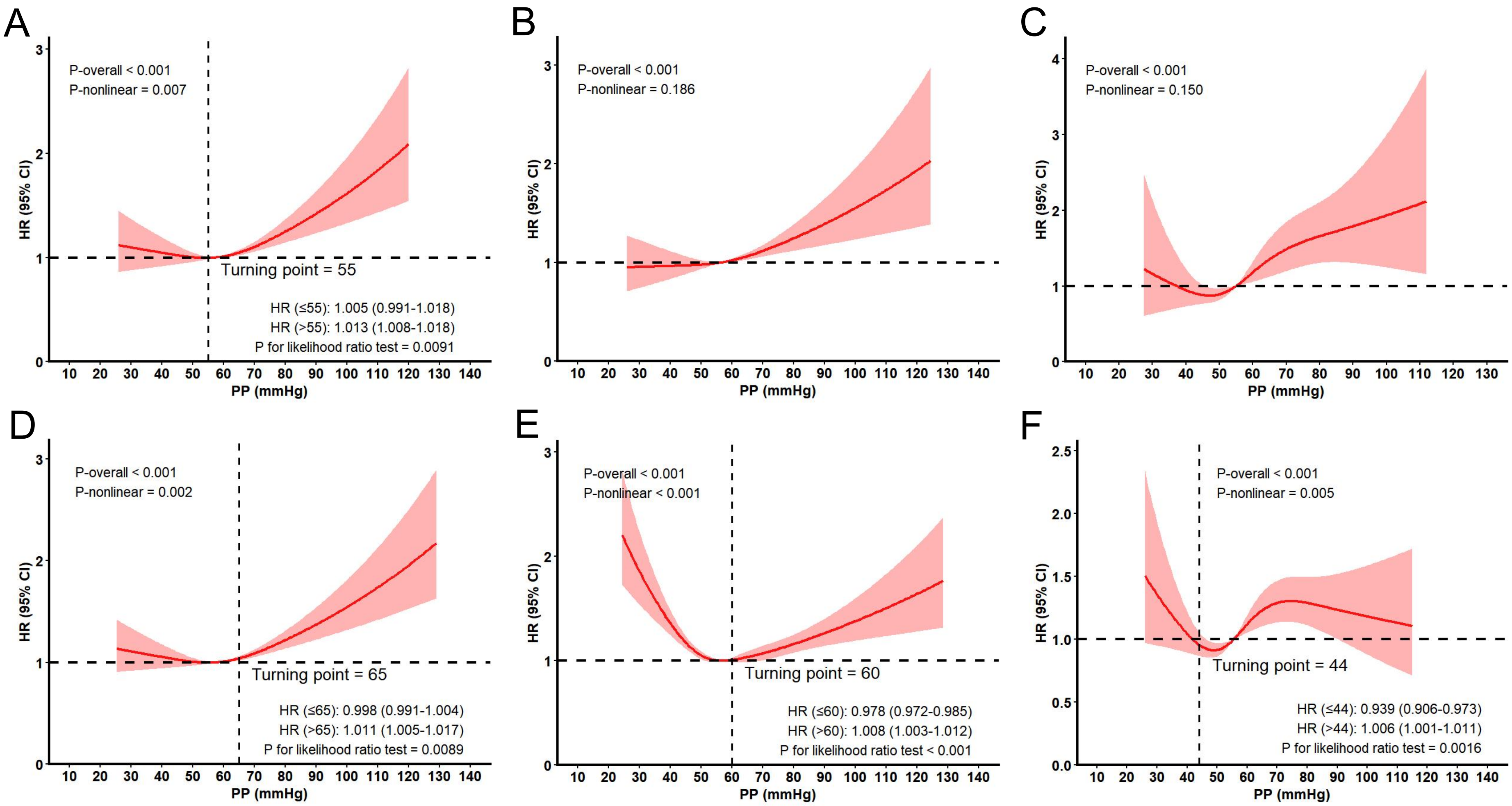

Figure S2. PP-associated transition from T2D/CHD/Stroke to CMM or mortality: RCS.

(A) T2D to CMM; (B) CHD to CMM; (C) Stroke to CMM; (D) T2D to all-cause mortality; (E) CHD to all-cause mortality; (F) Stroke to all-cause mortality. Abbreviations: CMM cardiometabolic multimorbidity, RCS restricted cubic splines, CHD coronary heart disease, T2D type 2 diabetes, RCS restricted cubic spline, PP pulse pressure.

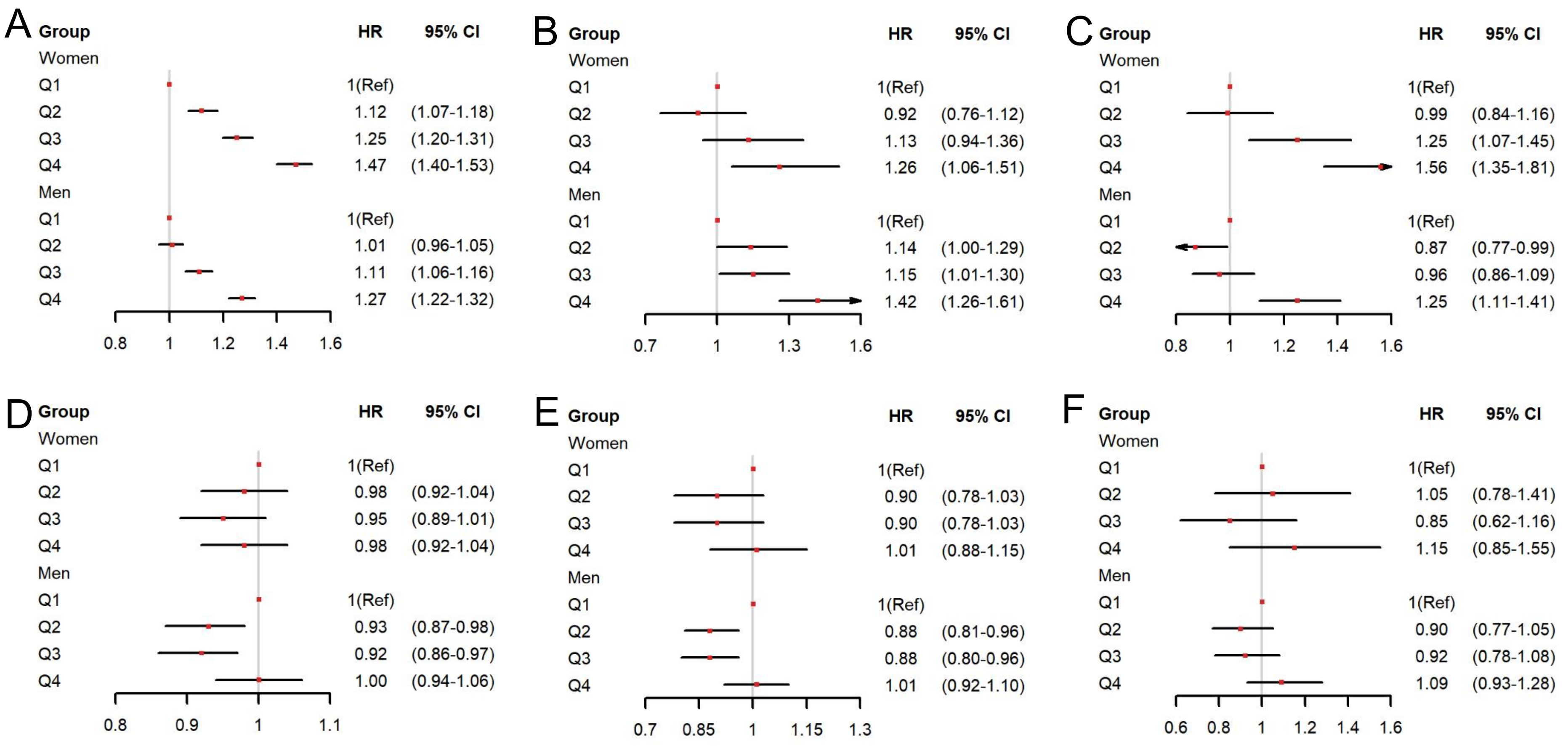

Figure S3. Subgroup analysis of PP-CMD/CMM-mortality associations by sex subgroups

(A) Healthy to one CMD; (B) one CMD to CMM; (C) Healthy to CMM; (D) Healthy to all-cause mortality; (E) one CMD to all-cause mortality; (F) CMM to all-cause mortality. Abbreviations: CMM cardiometabolic multimorbidity, CMD cardiometabolic diseases, Ref reference group, PP pulse pressure.

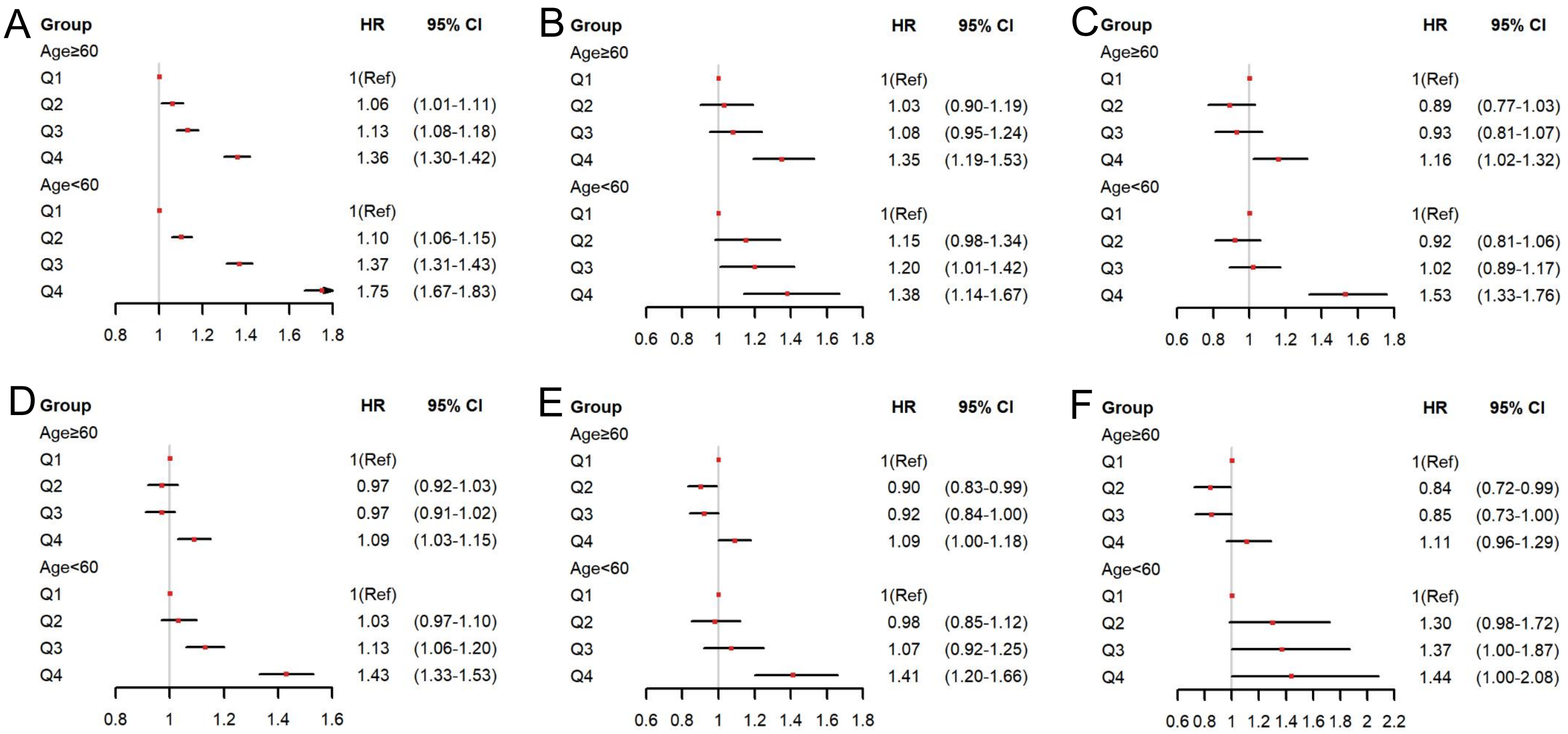

Figure S4. Subgroup analysis of PP-CMD/CMM-mortality associations by age subgroups

(A) Healthy to one CMD; (B) one CMD to CMM; (C) Healthy to CMM; (D) Healthy to all-cause mortality; (E) one CMD to all-cause mortality; (F) CMM to all-cause mortality. Abbreviations: CMM cardiometabolic multimorbidity, CMD cardiometabolic diseases, Ref reference group, PP pulse pressure.

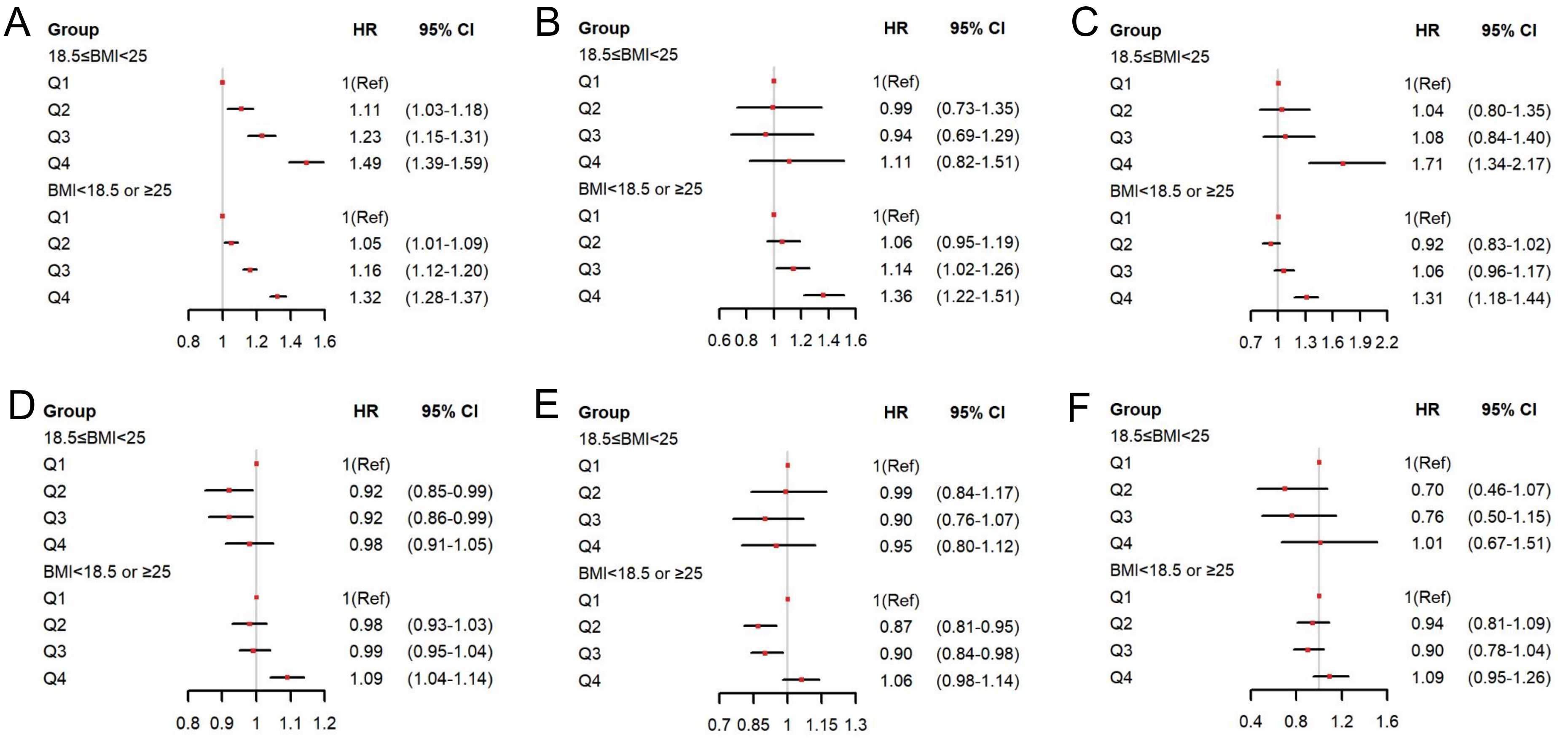

Figure S5. Subgroup analysis of PP-CMD/CMM-mortality associations by BMI category.

(A) Healthy to one CMD; (B) one CMD to CMM; (C) Healthy to CMM; (D) Healthy to all-cause mortality; (E) one CMD to all-cause mortality; (F) CMM to all-cause mortality. Abbreviations: CMM cardiometabolic multimorbidity, CMD cardiometabolic diseases, Ref reference group, PP pulse pressure, BMI body mass index.
